# Combination of Maternal Serum ESM-1 and PLGF with Uterine Artery Doppler PI for Predicting Preeclampsia

**DOI:** 10.3390/jcm12020459

**Published:** 2023-01-06

**Authors:** Xianjing Xie, Dan Chen, Xingyu Yang, Yunyun Cao, Yuna Guo, Weiwei Cheng

**Affiliations:** 1 International Peace Maternity and Child Health Hospital, School of Medicine, Shanghai Jiao Tong University, Shanghai 200030, China; 2 Shanghai Key Laboratory of Embryo Original Diseases, Shanghai 200030, China; 3 Department of Obstetrics and Gynecology, Renji Hospital, School of Medicine, Shanghai Jiao Tong University, Shanghai 200127, China; 4 Institute of Birth Defects and Rare Diseases, School of Medicine, Shanghai Jiao Tong University, Shanghai 200030, China

**Keywords:** preeclampsia, ESM-1, PLGF, uterine artery Doppler, PI, prediction

## Abstract

Objective: This study aimed to determine whether the combination of pregnancy-associated endothelial cell-specific molecule 1 (ESM-1), the placental growth factor (PLGF) in the first- and second-trimester maternal serum, and the uterine artery Doppler pulsatility index (PI) in the second trimester can predict preeclampsia (PE). Methods: The serum levels of ESM-1 and PLGF in 33 severe preeclampsia (SPE) patients, 18 mild preeclampsia patients (MPE), and 60 age-matched normal controls (CON) were measured. The Doppler ultrasonography was performed, and the artery pulsatility index (PI) was calculated for the same subjects. Results: The 2nd PLGF level was significantly lower and the 2nd PI was higher than those in the MPE group. Combining the 2nd PLGF with the 2nd PI yielded an AUC of 0.819 (83.33% sensitivity and 70.00% specificity). In the SPE group, the 1st ESM-1 level and the 2nd PLGF level were significantly lower, and the 2nd ESM-1 level and the 2nd PI were significantly higher in the SPE group. The combination of the 1st ESM-1, the 2nd PLGF, and the 2nd PI yielded an AUC of 0.912 (72.73% sensitivity and 95.00% specificity). Conclusions: The 1st ESM-1 and the 2nd PLGF levels and the 2nd PI were associated with PE. The combination of serum biomarkers and the PI improved the screening efficiency of the PE prediction, especially for SPE.

## 1. Introduction

Preeclampsia (PE) is a major cause of morbidity and mortality among pregnant women and infants, resulting in an estimated 76,000 maternal deaths and 500,000 fetal and newborn deaths every year [1]. The etiology of PE is still an enigma; currently, the most widely accepted theory for the development of PE is the “two-stage” theory. The first stage is reduced placental perfusion, and the second stage is generalized maternal endothelial dysfunction. Abnormal vascular growth and impaired endothelial function are considered to be the main components of the pathogenesis [2]. The imbalance between the antiangiogenic and proangiogenic factors is considered to be the link between the two stages. The expression of the antiangiogenic and angiogenic factors is altered in PE [3].

Endocan, an antiangiogenic factor which is also called endothelial cell-specific molecule-1 (ESM-1), was originally identified in cultured endothelial cells [4]. Several studies have shown that ESM-1 could be a novel biomarker of various diseases with endothelial dysfunction and inflammation, such as newly diagnosed hypertension [5]. A recent meta-analysis suggested that women with PE had a higher level of circulating ESM-1 than women with normal pregnancies [6].

Placental growth factor (PLGF), produced by villous syncytiotrophoblasts, is thought to induce nonbranching angiogenesis, leading to a low-resistance placental vascular network [7,8]. Limited angiogenesis in early PE pregnancies, with a shallow vascular invasion of the maternal spiral arteries, results in the subsequent placental hypoperfusion [9]. Many studies have revealed that angiogenic factors such as PLGF are decreased in the serum of PE patients [10,11]. PLGF has been proven to be a useful screening tool for PE prediction [12].

Spiral artery transformation failure in PE could lead to an increase in uterine artery blood flow resistance [13], which could be captured as an abnormality, such as by the presence of an impedance on the uterine artery Doppler. These changes support the uterine artery Doppler velocimetry-based screening of patients who are at risk of developing PE [14]. Furthermore, some studies have shown that uterine artery PI is a promising marker for predicting PE [15,16].

The objective of our study was to evaluate the maternal serum ESM-1 and the PLGF levels in the first and second trimesters and the uterine artery Doppler PI in the second trimester and to determine whether the integration of these biomarkers with the 2nd PI would be helpful in the prediction of PE.

## 2. Patients and Methods

### 2.1. Patients

This was a prospective study. Women with singleton pregnancies who presented at the International Peace Maternity and Child Health Hospital, Shanghai, China, from 2020 to 2021 for prenatal examination were eligible for inclusion. The exclusion criteria were multiple pregnancies, chronic hypertension, chronic renal disease or pre-existing proteinuria, diabetes, malignancy, autoimmune disorders, acute systemic inflammation, fever, premature rupture of membranes, preterm labor, or major congenital fetal anomaly. The study was conducted according to the Declaration of Helsinki guidelines and the approval of the National Ethics Committee for Science and Technology (number: GKLW2020-03). All the participants were followed from the first trimester to delivery, with pregnancy outcomes recorded and written informed consent provided.

The definition of the severe preeclampsia (SPE) group was as follows:

(1) Blood pressure ≥160/110 mm Hg on two occasions at least 4 h apart (unless antihypertensive therapy was initiated before this time);
(2) Thrombocytopenia: platelet count <100 × 10^9^/L;
(3) Renal insufficiency: serum creatinine concentrations >1.1 mg/dL or a doubling of the serum creatinine concentration in the absence of other renal diseases;
(4) Impaired liver function: elevated blood concentrations of liver transaminases to twice the normal concentration;
(5) Pulmonary edema;
(6) New-onset headache unresponsive to medication and not accounted for by alternative diagnoses or visual symptoms.

The mild preeclampsia (MPE) group was described as follows:

(1) Blood pressure ≥140/90 mmHg on two occasions at least 4 h apart after 20 weeks of gestation in a woman with previously normal blood pressure;
(2) Three hundred milligrams or more per 24 h of urine collection (or this amount extrapolated from a timed collection) or a protein/creatinine ratio ≥ 0.3 mg/dL or a dipstick reading of 2+.

### 2.2. Maternal Serum Analytes

Peripheral venous blood samples were collected from all the participants during two different periods: the first trimester, at 9–13^+6^ weeks of gestation, and the second trimester, at 24–28 weeks of gestation. All the blood samples were centrifuged at 3000 rpm for 10 min, and the serum samples were stored at −80 °C until use.

All the samples from the subsequently diagnosed PE patients based on the ACOG guidelines [17] and gestational age and storage time-matched control (CON) pregnancies were retrieved. The ESM-1 and PLGF levels were measured using the enzyme-linked immunosorbent assay (ELISA) (R&D Systems, Minneapolis, MN, USA) by technicians who were blinded to the identity of the samples. The accuracy and stability of the ELISA method were validated in the pilot experiments. Each sample was measured three times and the average level was used as the final value for the sample. The ESM-1 kit detection range was 10.3–2500 pg/mL. The kit performance characteristics were a sensitivity of 1.08 pg/mL and a coefficient of variation (CV%) of <10. The PLGF kit detection range was 2.88–700 pg/mL. The kit performance characteristics were a sensitivity of 1.9 pg/mL and a coefficient of variation (CV%) of <10.

### 2.3. Uterine Artery PI

The patients without fetal defects on routine ultrasound performed at 22–28 weeks of gestation also underwent a bilateral uterine artery Doppler assessment. The uterine artery PI was determined as the average PI from three continuous similar waveforms. All the examinations were evaluated by ultrasound by simultaneous B-mode scanning (GE Healthcare, Milwaukee, WI, USA). The carrier frequency was from 1 to 5 MHz for the transabdominal probers.

### 2.4. Statistical Analysis

The data analysis was performed using SPSS 25.0 (SPSS Inc., Chicago, IL, USA) and MedCalc (version 11.4.2.0). The data are presented as the mean ± SD or median (min-max). Logistic regression analysis was used to evaluate the combination of these indicators. Receiver operating characteristic (ROC) curve analysis was performed to assess the predictive value. Statistically significant differences were estimated utilizing the Student’s *t*-tests or chi-square tests. A *p* value < 0.05 was considered statistically significant.

## 3. Results

**Figure 1:**
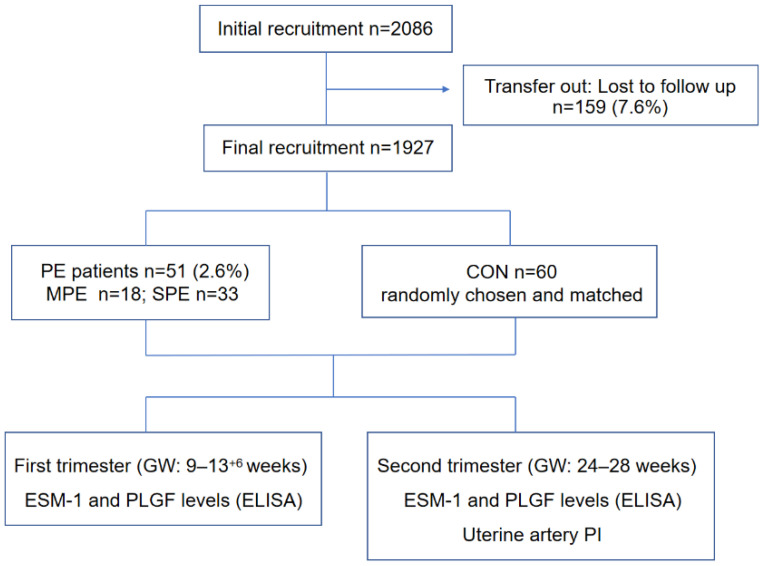
The flowchart of the prospective cohort study. CON, control group; MPE, mild preeclampsia group; SPE, severe preeclampsia group; GW, gestational week.

In total, 2086 pregnant women were recruited for the study. Among them, 1927 pregnant women completed the study, and 159 (7.6%) did not give birth in our hospital and were lost to the follow-up. Fifty-one women developed PE (severe preeclampsia (SPE) 33 cases and mild preeclampsia (MPE) 18 cases), with an incidence rate of 2.6%, which was consistent with that in the literature [18]. The CON group comprised 60 women with normal pregnancies who were randomly chosen and matched for gestational age to the women with PE. The flowchart of our prospective cohort study was shown in Figure 1.

The clinical and demographic characteristics of the participants are presented in Table 1. Compared with those in the CON group, the blood pressures of all those in the PE groups were higher (*p* < 0.001). The gestational age at delivery and the fetal weights were significantly lower in the PE group, especially in the SPE group (*p* < 0.001). The placental weights were lower in the SPE group (*p* < 0.05). However, there was no significant difference in maternal age, pre-pregnancy BMI, or the 5′ Apgar scores among the groups.

**Figure 2:**
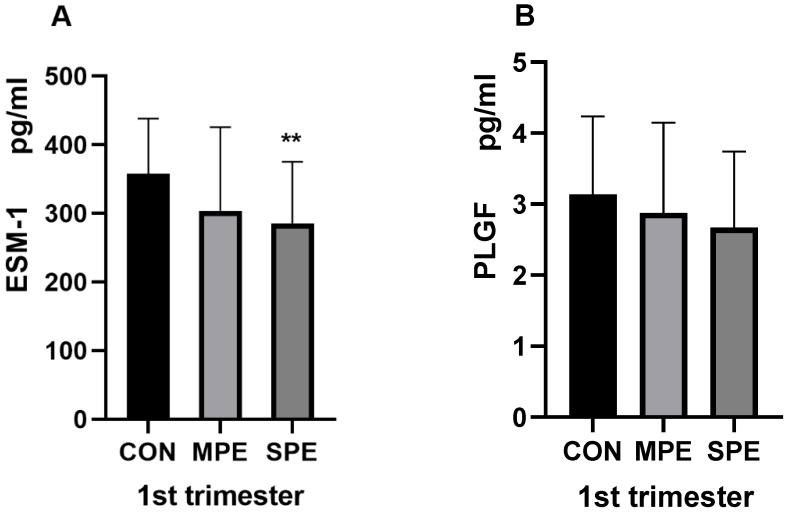
ESM-1 and PLGF in first-trimester maternal serum (**A**,**B**). CON, *n* = 60; MPE, *n* = 18; SPE, *n* = 33, ** *p* < 0.001.

**Figure 3:**
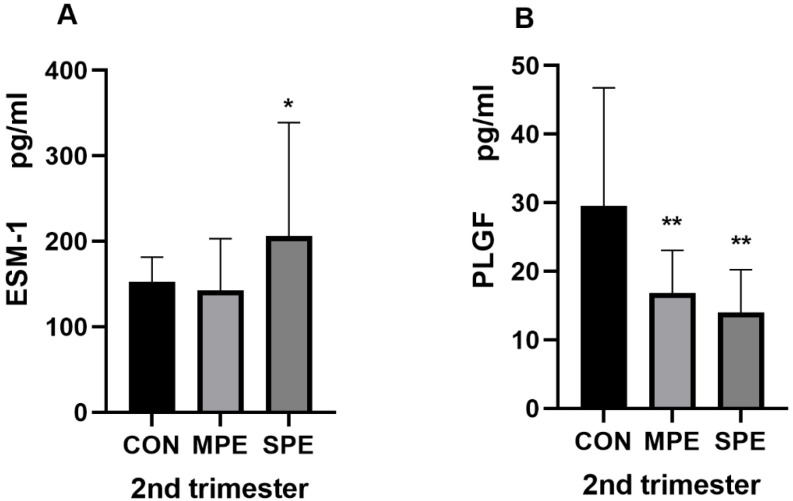
ESM-1 and PLGF in second-trimester maternal serum (**A**,**B**). CON, *n* = 60; MPE, *n* = 18; SPE, *n* = 33, * *p* < 0.05, ** *p* < 0.001.

**Figure 4:**
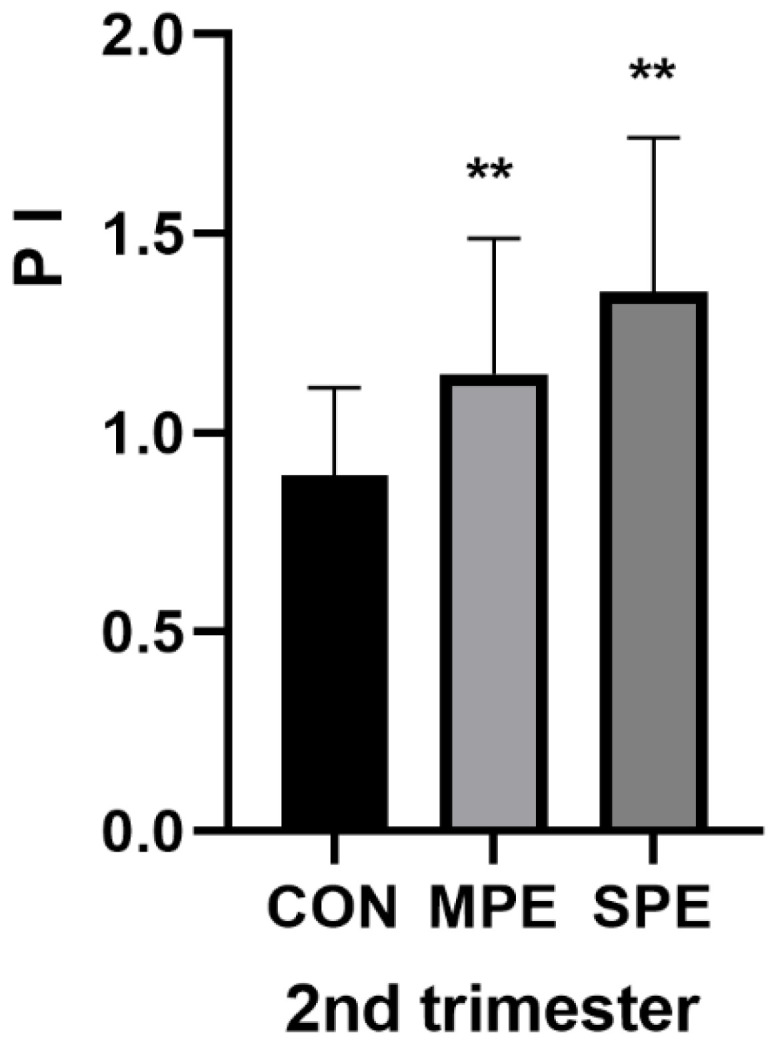
Uterine artery Doppler PI in the second trimester. CON, *n* = 60; MPE, *n* = 18; SPE, *n* = 33, ** *p* < 0.001.

The results of the serum analytes from the PE group and the CON group samples collected in the first and second trimesters are shown in Table 2 and Figure 2, Figure 3 and Figure 4. In the first trimester, compared with those in the gestational age-matched controls, the ESM-1 level (285.82 ± 89.53 vs. 357.61 ± 80.40, *p* < 0.001) in the SPE group was significantly lower. There was no significant difference in the ESM-1 level between the control and the MPE groups (Figure 2A). Additionally, there was also no significant difference in the PLGF level between the control group and any PE group (Figure 2B). In the second trimester, compared with those in the gestational age-matched controls, the ESM-1 level (206.24 ± 132.53 vs. 152.35 ± 29.00, *p* = 0.0032) in the SPE group was significantly higher and the PLGF level (14.03 ± 6.21 vs. 29.52 ± 17.26, *p* < 0.001) in the SPE group was significantly lower (Figure 3A,B). Moreover, compared to those in the control group, the PLGF level was significantly lower (16.82 ± 6.25 vs. 29.52 ± 17.26, *p* < 0.001) in the MPE group (Figure 3B). There was no significant difference in the ESM-1 level between the control and the MPE groups (Figure 3A). Compared with those in the gestational age-matched controls, the 2nd PI (1.35 ± 0.39 vs. 0.89 ± 0.22, *p* < 0.001) in the SPE group and the 2nd PI (1.15 ± 0.34 vs. 0.89 ± 0.22, *p* < 0.001) in the MPE group were also significantly higher (Figure 4).

**Figure 5:**
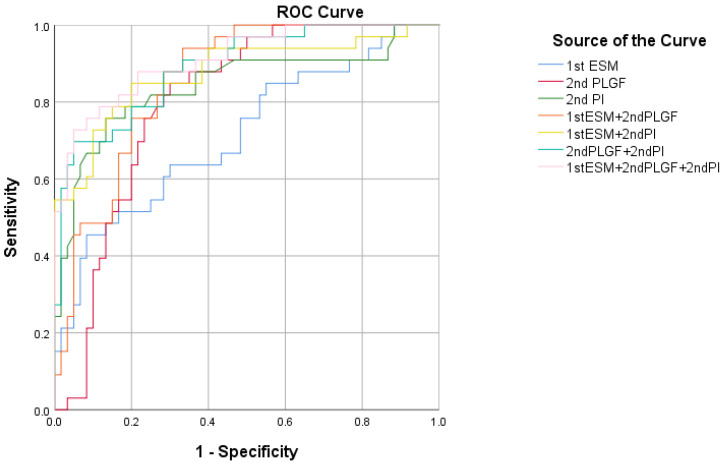
Receiver operating characteristic curve showing the clinical discrimination of different markers alone or in combination in the detection of severe preeclampsia.

Furthermore, the details of the AUCs are shown in Table 3, and the ROC curves for the biomarkers in the SPE prediction are presented in Figure 5. The ROC analysis for the SPE and control subjects yielded AUCs for the 1st ESM-1, the 2nd PLGF, and the 2nd PI of 0.714 (95% CI: 0.611–0.803, *p* = 0.0002), 0.802 (95% CI: 0.706–0.877, *p* < 0.0001), and 0.843 (95% CI: 0.753–0.911, *p* < 0.0001), respectively. Logistic regression analysis was used to evaluate the combination of these indicators. In the SPE group, the AUCs for the combinations of the 1st ESM-1 and 2nd PLGF, the 1st ESM-1 and 2nd PI, and the 2nd PLGF and 2nd PI were 0.856 (93.90% sensitivity and 66.70% specificity), 0.876 (84.85% sensitivity and 80.00% specificity), and 0.890 (69.70% sensitivity and 95.00% specificity), respectively. The combination of the 1st ESM-1, the 2nd PLGF, and the 2nd PI yielded an AUC of 0.912 (72.73% sensitivity and 95.00% specificity) (*p* < 0.0001 for all). 

**Figure 6:**
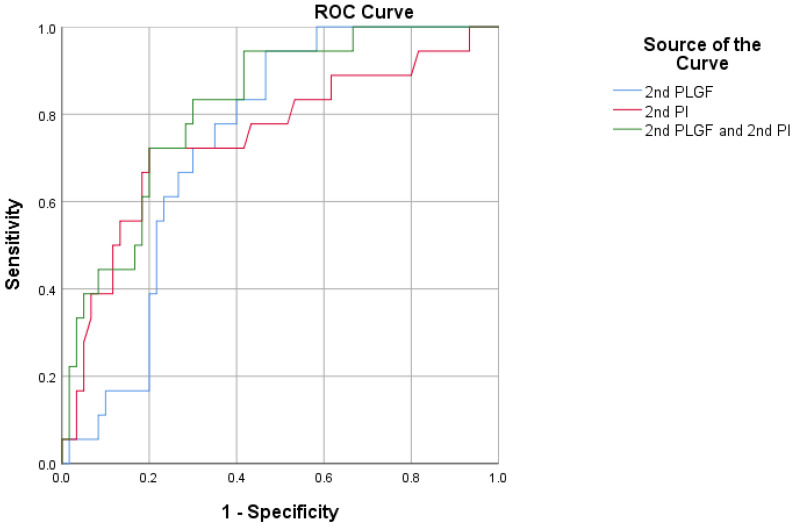
Receiver operating characteristic curve showing the clinical discrimination of different markers alone or in combination in the detection of mild preeclampsia.

The ROC curves for the biomarkers in the MPE prediction are presented in Figure 6. The ROC analysis for the MPE and control subjects yielded areas under the curve (AUCs) for the 2nd PLGF and the 2nd PI of 0.738 (95% CI: 0.626–0.831, *p* < 0.0001) and 0.748 (95% CI: 0.636–0.839, *p* = 0.0007), respectively. In the MPE group, combining the 2nd PLGF with the 2nd PI yielded an AUC of 0.819 (83.33% sensitivity and 70.00% specificity).

## 4. Discussion

Accumulative evidence has revealed that inflammation and endothelial dysfunction are vitally important to the pathophysiology of PE [19]. ESM-1 might be involved in endothelial-related processes, including cell adhesion, angiogenesis, inflammation, and endothelial dysfunction [20]. Thus, ESM-1 could be regarded as a biomarker for hypertension [5,21], sepsis [22], malignancy [23], and PE [6].

The results showed that the ESM-1 level in the maternal plasma was lower during the first trimester (12–16 weeks of gestation) but increased in the second and third trimesters (≥24 weeks of gestation) in the SPE group [24]. Some studies in 2015 and 2016 reported higher ESM-1 concentrations in PE maternal plasma and a negative correlation with the clinical data, indicating its crucial role in the pathogenesis of PE progression [25,26]. Moreover, the stratified results from a meta-analysis conducted to determine the potential role of ESM-1 in PE suggested the upregulation of ESM-1 levels in PE [6]. The results obtained in our study were consistent with these studies, except that ESM-1 was detected earlier in the first trimester in our study.

As an explanation for the lower 1st ESM-1 level in PE, some researchers have suggested that ESM-1 functions as a protective cytokine by inhibiting leukocyte aggregation to protect tissues and organs from inflammatory damage and is consumed in early pregnancy [25]. It was also indicated that a positive feedback loop exists between the vascular endothelial growth factor (VEGF) and ESM-1 [27]; therefore, as the results revealed, the 2nd ESM-1 level was reduced with the advancement of the placental vasculature in the CON group and PE groups. In addition, ESM-1 can be upregulated by proinflammatory factors and growth factors, such as TNF-α, IL-6, and VEGF [28]. With the development of PE, the inflammatory response and endothelial dysfunction were aggravated, and the ESM-1 level was elevated in the second and third trimesters. Regarding the lack of a significant difference in the ESM-1 level between the MPE group and the normal pregnancy group in the first and second trimesters, it was speculated that inflammation and endothelial dysfunction were too minor to distinguish.

PLGF, which is expressed in the human placenta, heart, and lungs, is a member of the VEGF family [29]. It can directly activate its angiogenic pathway through PLGF/VEGFR1 (known as FLT1) and compete with VEGF-A for VEGFR1, further stimulating angiogenesis via the VEGFA/VEGFR2 (known as FLK1) interaction. During normal pregnancy, the PLGF level is relatively low in the first trimester. It gradually increases with the advancement of the utero-placental circulation and remodeling of the myometrial spiral arteries and finally reaches its peak at approximately 30 weeks of gestation, after which it drops [8]. Given the vital role of the placenta in PE, the usefulness of PLGF in PE prediction was investigated. A previous study revealed that at 9–12^+6^ weeks of gestation, the PLGF expression was significantly lower in PE pregnancies than in the CON pregnancies [30]. Another study examined the concentrations of PLGF in women with PE during two periods (8–14 weeks of gestation and 20–34 weeks of gestation). The results suggested that the PLGF level in the PE group was significantly lower in the first period, but no difference was found in the second period between the PE group and the CON group [31]. The study investigated the remarkably lower PLGF level in PE pregnancies at 24–28 weeks of gestation [32], and another study found that the PLGF concentration was also significantly lower before 35 weeks in the PE pregnancy compared to the normal pregnancy [33]. Moreover, the PLGF change was relevant to disease severity [34]. Our study had similar results. Specifically, a lack of a significant difference in the 1st PLGF level was found between the PE group and the CON group. Additionally, the 2nd PLGF concentrations were elevated compared to the 1st PLGF concentrations, but they were significantly lower than the 2nd PLGF concentration in the CON group, especially for SPE. With pregnancy progression in PE, the PLGF might compete with the VEGF for binding to sFlt-1, resulting in a significant drop emerging in the second trimester before the onset of PE symptoms [8].

Some studies have shown that uterine artery PI is a significant marker for predicting PE. The Doppler ultrasound of the maternal uterine artery PI might be the most effective method for screening women with PE in the second trimester [35]. In our study, 75.76% sensitivity and 86.67% specificity were achieved with the uterine artery PI. The uterine artery PI was increased in the second-trimester pregnancies in the PE patients, especially the SPE patients, which was consistent with the reported literature.

Many recent studies have suggested that the combination of biochemical indicators and the uterine artery Doppler could improve the screening efficiency for the prediction of PE [36,37]. Notably, our study similarly indicated that the overall predictive efficiency for SPE achieved by combining serum biomarkers and the uterine artery Doppler PI was improved compared with the single use of any marker alone.

## 5. Conclusions

The 1st ESM-1, 2nd PLGF levels and the 2nd uterine artery Doppler PI were associated with PE. The combination of serum biomarkers and the uterine artery Doppler PI strengthened the screening efficiency for the prediction of PE, especially for SPE. Our study is the first to assess the predictive combination of the 1st ESM-1, the 2nd PLGF, and the 2nd uterine artery Doppler PI for PE. Unfortunately, due to the COVID-19 outbreak, the blood samples of the participants in the third trimester were lost, resulting in a lack of data on the serum concentrations of ESM-1 and PLGF. Further studies are needed in a larger population to determine the potential for use of the aforementioned indicators.

## Figures and Tables

**Table 1:** Demographic characteristics of the participants.

Characteristic	SPE (*n* = 33)	MPE (*n* = 18)	CON (*n* = 60)
Maternal age, y	31.06 ± 4.34	31.89 ± 4.38	30.03 ± 3.61
Pre-BMI, kg/m^2^	21.02 ± 2.55	21.51 ± 2.02	20.60 ± 1.82
SBP (mmHg)	166.18 ± 9.74 **	144.39 ± 5.38 **	117.00 ± 9.39
DBP (mmHg)	97.91 ± 8.91 **	92.33 ± 5.24 **	74.78 ± 9.11
Nulliparous, n (%)	29 (87.88%)	16 (88.89%)	52 (86.67%)
GA at delivery, weeks	37.09 ± 3.01 **	38.73 ± 1.20 *	39.45 ± 0.81
Fetal weight, g	2660.45 ± 754.43 **	3161.39 ± 266.34 *	3375.50 ± 297.29
Placental weight, g	537.27 ± 165.52 *	622.50 ± 84.53	614.08 ± 67.01
5′ Apgar score	9.33 ± 1.83	9.61 ± 0.61	9.81 ± 0.62

SPE, severe preeclampsia; MPE, mild preeclampsia; CON, control; pre-BMI: pre-pregnancy body mass index; SBP, systolic blood pressure; DBP, diastolic blood pressure; GA, gestational age. * *p* < 0.05; ** *p* < 0.001 compared to the CON.

**Table 2:** Comparison of biomarker levels among study groups.

	SPE (*n* = 33)	MPE (*n* = 18)	CON (*n* = 60)	*p* Value	
				SPE	MPE
1st ESM-1 (pg/mL)	285.82 ± 89.53	304.00 ± 121.59	357.61 ± 80.40	<0.001 **	0.093
2nd ESM-1 (pg/mL)	206.24 ± 132.53	142.58 ± 60.68	152.35 ± 29.00	0.0032 *	0.347
1st PLGF (pg/mL)	2.67 ± 1.07	2.87 ± 1.27	3.13 ± 1.10	0.0524	0.400
2nd PLGF (pg/mL)	14.03 ± 6.21	16.82 ± 6.25	29.52 ± 17.26	<0.001 **	<0.001 **
2nd PI	1.35 ± 0.39	1.15 ± 0.34	0.89 ± 0.22	<0.001 **	<0.001 **

SPE, severe preeclampsia; MPE, mild preeclampsia; CON, control; ESM-1, endothelial cell-specific molecule 1; PLGF, placental growth factor; PI, pulsatility index. 1st/2nd ESM-1, ESM-1 level measured in the first/second trimester; 1st/2nd PLGF, PLGF level measured in the first/second trimester; 2nd PI, PI measured in the second trimester. * *p* < 0.05; ** *p* < 0.001 compared to the CON.

**Table 3:** Predictive efficiency of biomarker levels and PI for severe preeclampsia.

Variable	AUC	*p* Value	95%CI	Cutoff	Specificity (%)	Sensitivity (%)
1st ESM-1	0.714	0.0002	0.611–0.803	262. 33	91.67	45.50
2nd PLGF	0.802	<0.0001	0.706–0.877	19.12	70.00	84.85
2nd PI	0.843	<0.0001	0.753–0.911	1.12	86.67	75.76
1st ESM-1 + 2nd PLGF	0.856	<0.0001	0.767–0.920	-	66.70	93.90
1st ESM-1 + 2nd PI	0.876	<0.0001	0.792–0.935	-	80.00	84.85
2nd PLGF + 2nd PI	0.890	<0.0001	0.808–0.945	-	95.00	69.70
1st ESM-1 + 2nd PLGF + 2nd PI	0.912	<0.0001	0.835–0.961	-	95.00	72.73

CI: confidence interval; ESM-1, endothelial cell-specific molecule 1; PLGF, placental growth factor; PI, pulsatility index; 1st/2nd ESM-1, ESM-1 level measured in the first/second trimester; 1st/2nd PLGF, PLGF level measured in the first/second trimester.

## Data Availability

All the study data will be made available upon request to the corresponding author.
